# Application Value of Gastroenterography Combined With CT in the Evaluation of Short-Term Efficacy and Prognosis in Patients With Esophageal Cancer Radiotherapy

**DOI:** 10.3389/fsurg.2022.898965

**Published:** 2022-06-09

**Authors:** Liangliang Xue, Linning E, Zhifeng Wu, Dongqiang Guo

**Affiliations:** Department of Radiology, Shanxi Bethune Hospital, Shanxi Academy of Medical Sciences, Taiyuan, China

**Keywords:** esophageal cancer, radiotherapy, gastroenterography, CT, short-term efficacy, prognosis

## Abstract

**Purpose:**

To observe the application value of gastroenterography combined with CT in the evaluation of short-term efficacy and prognosis in patients with esophageal cancer radiotherapy.

**Methods:**

From January 2013 to December 2020, the clinical data of 207 patients with esophageal cancer treated by radiotherapy in our hospital were collected retrospectively. All patients received gastroenterography and CT examination before and after radiotherapy, and the patients were followed-up for 1 year, and the evaluation value of their short-term efficacy and prognosis was observed.

**Results:**

After radiotherapy, the length diameter, short diameter, and volume of the lymph node were lower than those before radiotherapy (*p* < 0.05), but the maximum tube wall thickness had no significant difference (*p* > 0.05). The length diameter, short diameter, and volume of the lymph node, and the maximum tube wall thickness in the good efficacy group and the good prognosis group were lower, and the objective response rate in the good prognosis group was higher (*p* < 0.05). The area under the curve (AUC) of the length diameter, short diameter, and volume of the lymph node to evaluate the short-term efficacy of patients with esophageal cancer was 0.738, 0.705, and 0.748, respectively, and the AUC to evaluate the prognosis of patients with esophageal cancer was 0.751, 0.776, and 0.791, respectively.

**Conclusion:**

Gastroenterography combined with CT has a good application value in the evaluation of short-term efficacy and prognosis in patients with esophageal cancer radiotherapy.

## Introduction

The main manifestations of esophageal cancer are damage to the esophageal wall and narrowing of the esophageal cavity. Lesions will involve the submucosa, leading to muscular hyperplasia, which affects patients' esophageal diastolic function (1). In recent years, with the changes in people's lifestyles and eating habits, the number of patients with esophageal cancer is increasing. The disease has a serious impact on the life safety of patients and has become the main disease endangering human health. According to the report, in 2020, esophageal cancer ranks eighth (600,000) in the global cancer incidence, and ranks sixth (540,000) in the number of deaths. Furthermore, in 2020, esophageal cancer ranks sixth (320,000) in China's cancer incidence, and it ranks fourth (300,000) in the number of deaths (2). Therefore, early treatment of patients with esophageal cancer can effectively improve the prognosis of patients. Once the patients with esophageal cancer are diagnosed, only about 20% of patients can be treated by radical resection, and about 80% of patients need radiotherapy or other methods to relieve the disease (3). Radiotherapy is one of the commonly used methods to treat esophageal cancer. This method can significantly reduce the volume of the lesion, create favorable conditions for further treatment of patients, and is of great significance for improving the prognosis of patients (4). However, some patients still have local and regional recurrence after radiotherapy, which leads to treatment failure. If the short-term efficacy of radiotherapy in patients with esophageal cancer can be scientifically and accurately evaluated, and prognosis evaluation can be carried out, it will be beneficial to improve the quality of life of patients. At present, with the continuous development of imaging technology, more and more physicians choose evaluation tools, such as CT and X-ray gastroenterography to comprehensively evaluate the curative effect and prognosis of patients with esophageal cancer (5). However, the use of a single tool to evaluate the disease status of patients with esophageal cancer has many limitations. Therefore, we aimed to observe the application value of gastroenterography combined with CT in the evaluation of short-term efficacy and prognosis in patients with esophageal cancer radiotherapy.

## Materials and Methods

### Research Object

From January 2013 to December 2020, the clinical data of 207 patients with esophageal cancer treated by radiotherapy in our hospital were collected retrospectively. Inclusion criteria: it was esophageal cancer, as confirmed by pathological puncture; received radiotherapy for the first time; there were no contraindications of radiotherapy; the patient was able to take care of themselves; and the basic information was complete. Exclusion criteria: the lesion had metastasized to a distant place; Karnofsky score <70 points; combined with other malignant tumors; there were metabolic diseases; there were blood diseases; there were autoimmune diseases; existence of mental illness; and there were important organ injuries. Among 207 patients, there were 113 men and 94 women, with an average age of (59.16 ± 3.27) years. Pathological classification: 185 cases of squamous cell carcinoma, 19 cases of adenocarcinoma, and 3 cases of small cell carcinoma; clinical staging: 28 cases in stage I, 105 cases in stage II, and 74 cases in stage III; location of lesions: 15 cases of the cervical segment (cricoid cartilage—thoracic cavity entrance), 50 cases of the upper thoracic segment (thoracic cavity entrance—tracheal bifurcation), 118 cases of a middle thoracic segment (tracheal bifurcation—the upper half of the full length of the esophagogastric junction), and 24 cases of the lower thoracic segment (lower half of the full length of the esophagogastric junction).

### Research Methods

① Gastroenterography examination: medical staff instructed the patient to be in an upright position, and orally took 65 ml air barium suspension. Multi-position radiography was used to determine the lesion scope.
② Imaging examinations before radiotherapy: gastroenterography and CT examination were performed using a CT scanner [SOMATOM Definition AS (SIEMENS)]. The medical staff instructed patients to lie in the supine position and instruct patients to breathe evenly in a calm condition. The scanning layer thickness was 3 mm, and the layer spacing was set to 3 mm. The neck, upper abdomen, and chest of a patient were scanned, and the scan should reach the lower part of the kidney from the entrance of the esophagus. Medical staff reconstructs 3D images by scanning images. The location of the lesions was determined according to the results of imaging examinations, such as gastroenterography, CT, and fiberoptic esophagoscopy. Criteria for the location of lesions are: the thickness of esophageal wall >5 mm, the excluded diameter of tracheal lumen > 10 mm, thickening of the esophageal wall, and/or local stenosis of the esophageal lumen. The dose of radiotherapy was 54–69 or 1.8–2.0 Gy/time, and patients need to be treated with radiotherapy 5 times a week.
③ Imaging examination after radiotherapy: the interval between layers was set to 6 mm. The patient's neck to the upper abdomen was scanned. According to the location of the lesion known before radiotherapy, the location of the original lesion on the CT scan image after radiotherapy can be determined. The maximum tube wall thickness was measured, and the length diameter, short diameter, and volume of lymph nodes before and after radiotherapy were determined.
④ The short-term efficacy of gastroenterography was evaluated after 3 months of radiotherapy according to the esophageal cancer practice guidelines (6). Complete response (CR): the lesion completely disappears, barium can completely pass through, the esophageal lumen has slight stenosis or no stenosis, the esophageal mucosa is thickened, and the tube wall is slightly stiff; partial response (PR): the lesion disappeared by ≥30%, no extra-cavity ulcer, and esophageal distortion were found, barium could pass completely, the edge of the esophagus was not smooth enough, and small niches could be observed; stable disease (SD): the lesion is not significantly reduced, the esophageal filling defect is significant, and the lumen stenosis is serious; and progressive disease (PD): obvious new lesions were observed. Objective response rate (ORR) = CR+PR/total cases × 100%.
⑤ After radiotherapy, the medical staff told the patients to follow-up regularly. They followed-up patients for 1 year, and their survival rate was recorded.

### Statistical Methods

In this study, SPSS 22.0 was used for data processing. The measurement data that conform to the normal distribution were expressed by mean ± standard deviation (SD), and the comparison was made by *t*-test. The count data were expressed by percentage, and the comparison was made by the χ^2^ test. The receiver operating characteristic curve (ROC) was used to analyze the evaluation value of imaging results. The value of *p* < 0.05 was statistically significant.

## Results

### Comparison of Imaging Results Before and After Radiotherapy

**Figure 1:**
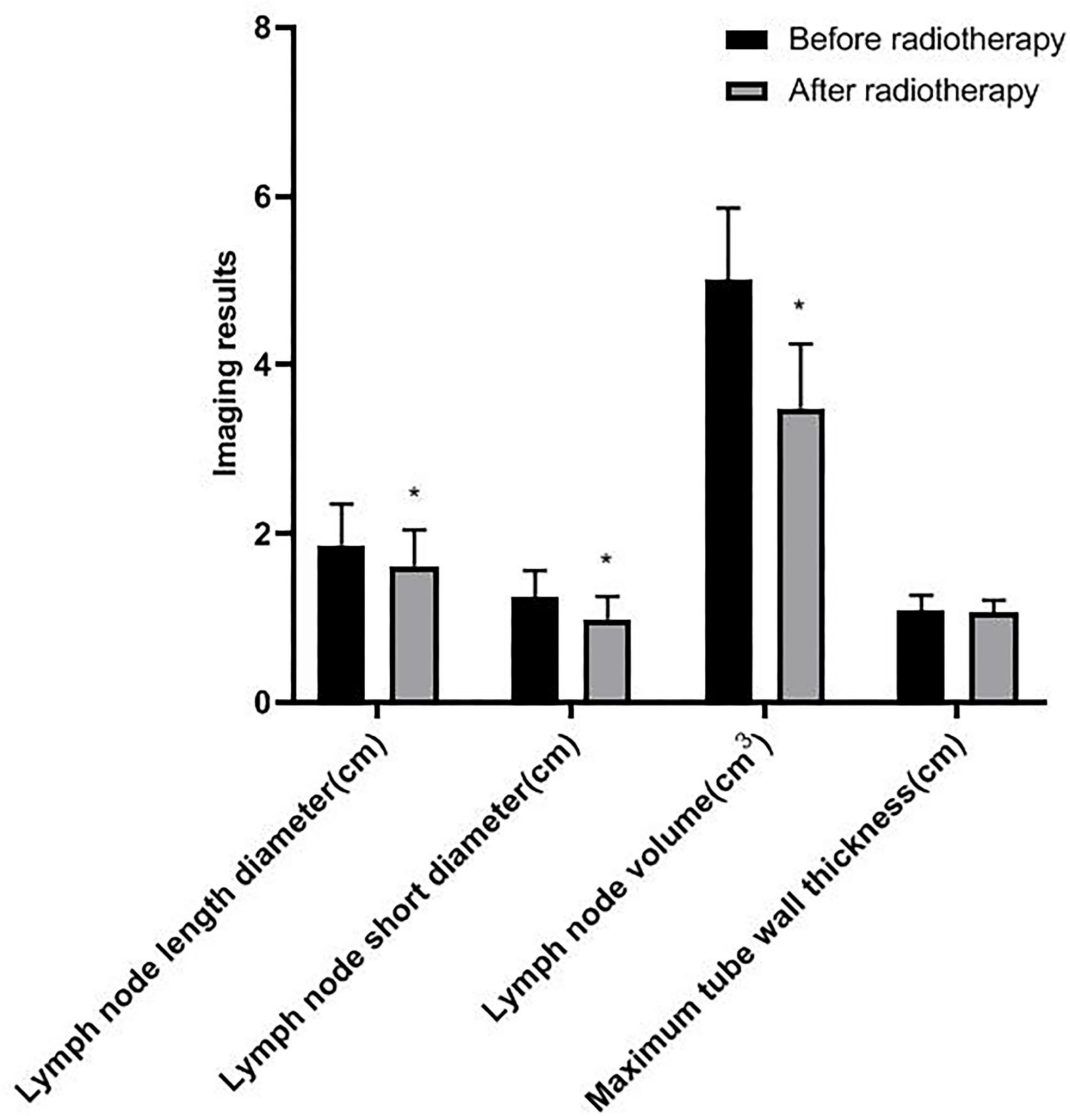
Comparison of imaging results before and after radiotherapy. Compared with before radiotherapy, *p < 0.05.

After radiotherapy, the length diameter, short diameter, and volume of the lymph node were lower than those before radiotherapy (*p* < 0.05), but the maximum tube wall thickness had no significant difference (*p* > 0.05) (as shown in Figure 1).

### Comparison of Imaging Results of Patients With Different Efficacy

**Figure 2:**
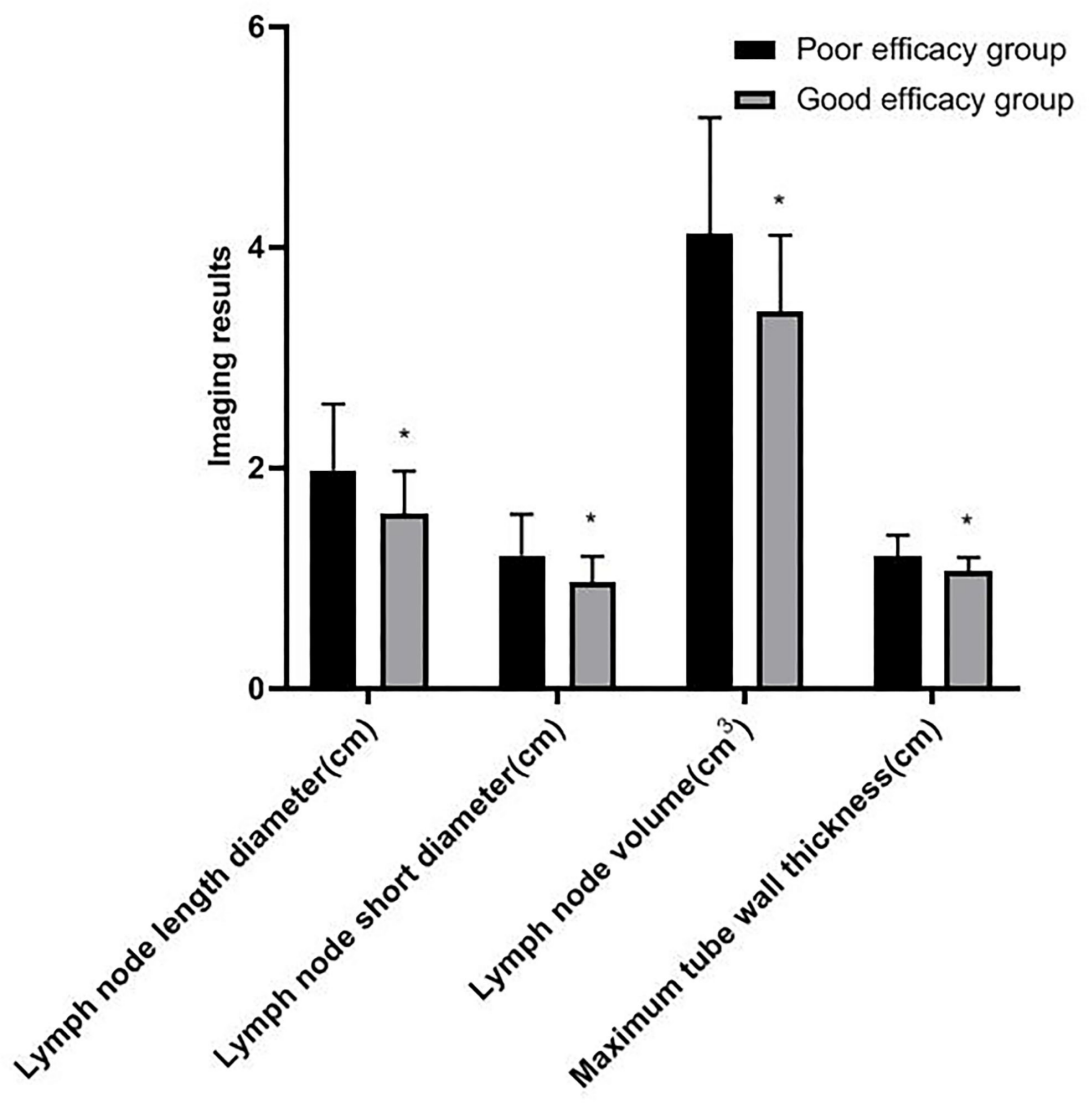
Comparison of imaging results of patients with different efficacy. Compared with the poor efficacy group, *p < 0.05.

Among 207 patients with esophageal cancer treated by radiotherapy, CR was 62 cases, PR was 124 cases, SD was 18 cases, PD was 3 cases, and ORR was 89.85% (186/207). After radiotherapy, the patients were divided into two groups: the good efficacy group (186 cases) and the poor efficacy group (21 cases). The length diameter, short diameter, and volume of lymph nodes and the maximum tube wall thickness in the good efficacy group were lower than those in the poor efficacy group (*p* < 0.05) (as shown in Figure 2).

### Comparison of Imaging Results of Patients With Different Prognosis

**Figure 3:**
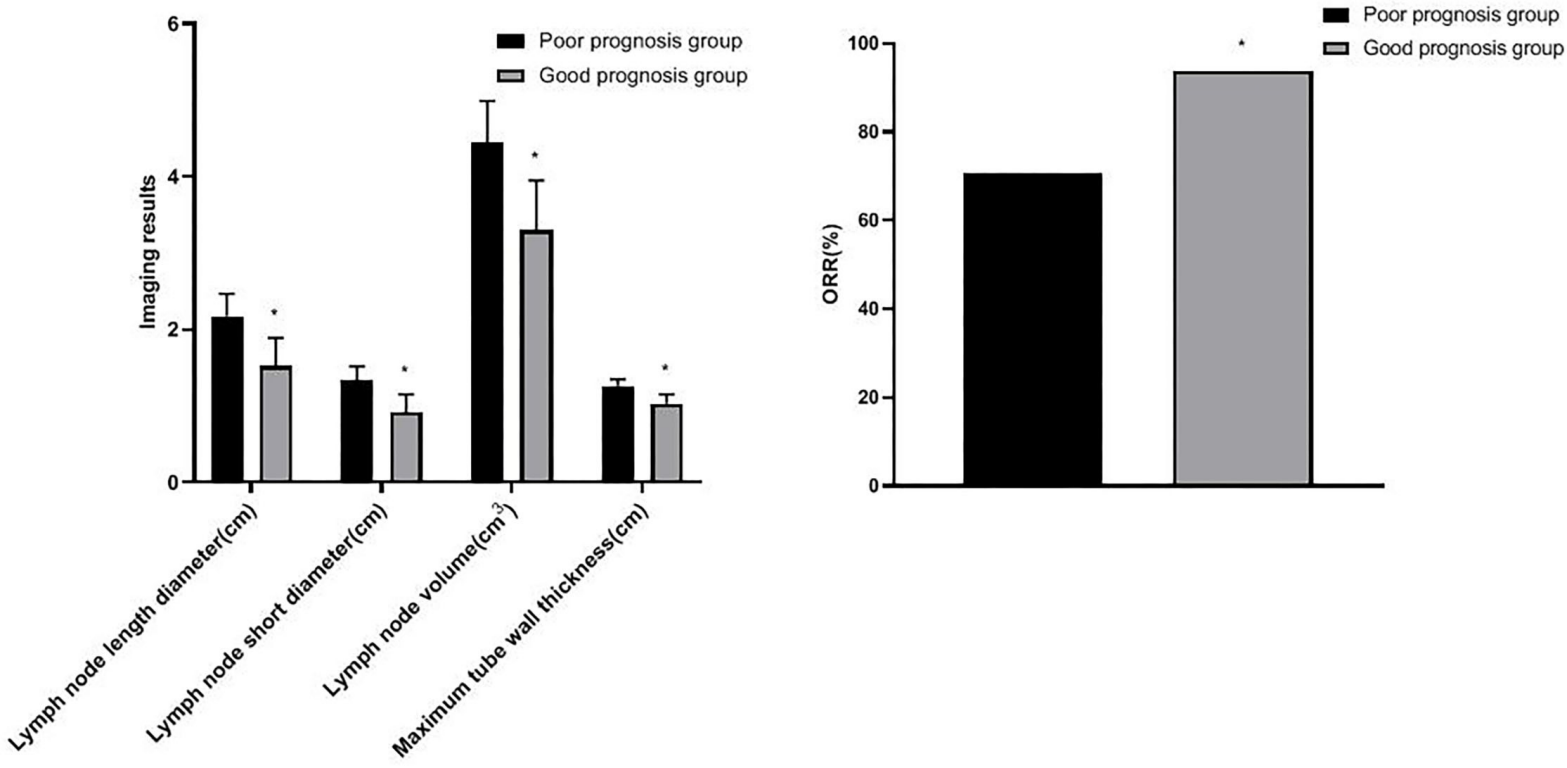
Comparison of imaging results of patients with different prognosis. Compared with the poor prognosis group, *p < 0.05.

After radiotherapy, the patients were followed-up for 1 year. Among 207 patients with esophageal cancer treated by radiotherapy, 173 cases survived and 34 cases died, and the survival rate was 83.57% (173/207). Patients were divided into two groups: the good prognosis group (173 cases) and the poor prognosis group (34 cases). The length diameter, short diameter, and volume of lymph nodes and the maximum tube wall thickness in the good prognosis group were lower than those in the poor prognosis group, and the ORR of the good prognosis group (93.64%) was higher than the poor prognosis group (70.59%) (*p* < 0.05) (as shown in Figure 3).

### Value of Imaging Results in Evaluating Short-Term Efficacy

**Figure 4:**
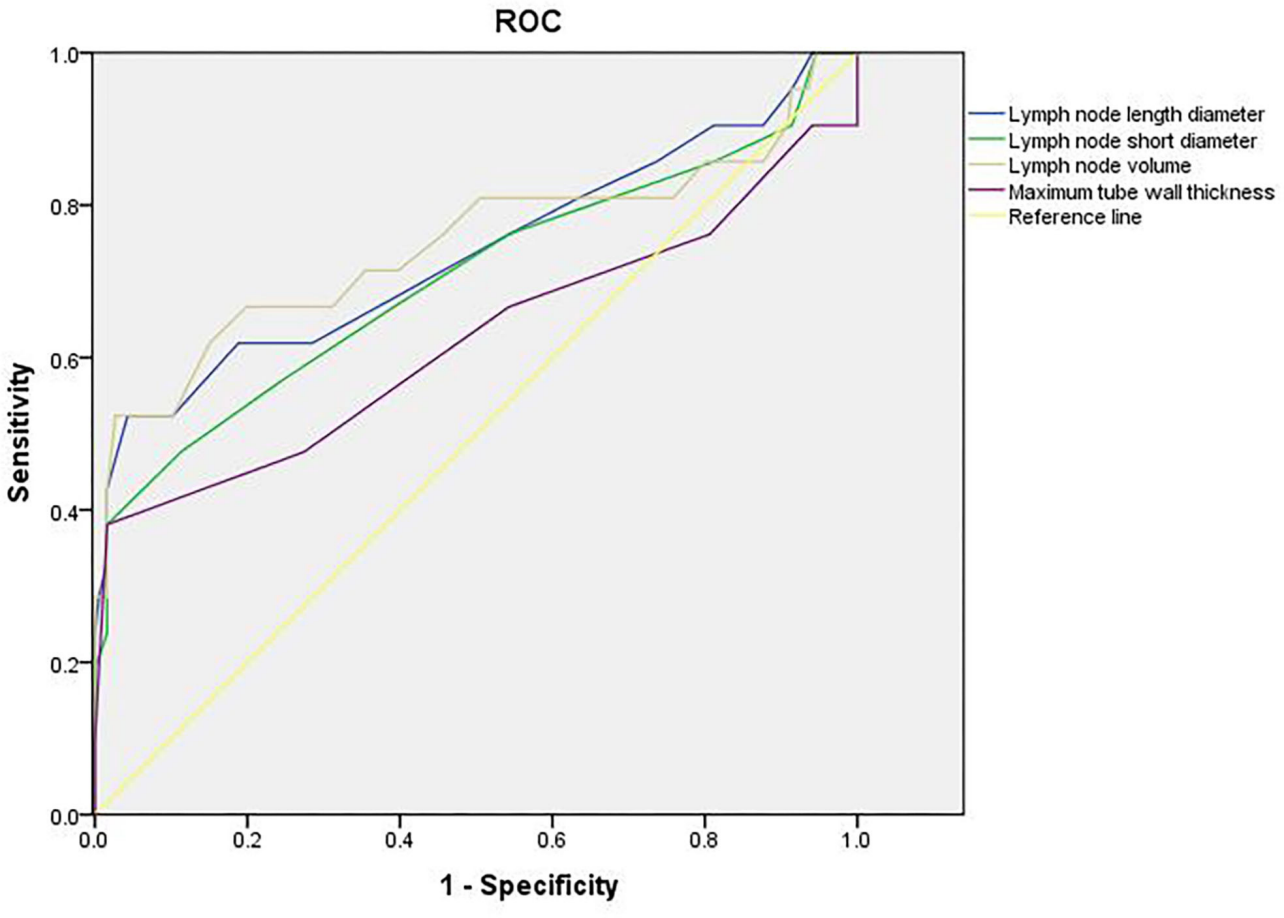
Value of imaging results in evaluating short-term efficacy.

The area under the curve (AUC) of length diameter, short diameter, and volume of the lymph node in evaluating the short-term efficacy of esophageal cancer patients was 0.738, 0.705, and 0.748, respectively (*p* < 0.05). The AUC of maximum tube wall thickness in evaluating the short-term efficacy of esophageal cancer patients was 0.622 (*p* > 0.05) (as shown in Table 1 and Figure 4).

**Table 1:** Value of imaging results in evaluating short-term efficacy.

**Index**	**AUC**	**Asymptotic 95% confidence interval**		**Standard error**	***P*-value**
		**Lower limit**	**Upper limit**		
Lymph node length diameter	0.738	0.592	0.879	0.072	0.000
Lymph node short diameter	0.705	0.561	0.849	0.074	0.002
Lymph node volume	0.748	0.603	0.894	0.074	0.000
Maximum tube wall thickness	0.622	0.459	0.784	0.083	0.068

### Value of Imaging Results in Evaluating Prognosis

**Figure 5:**
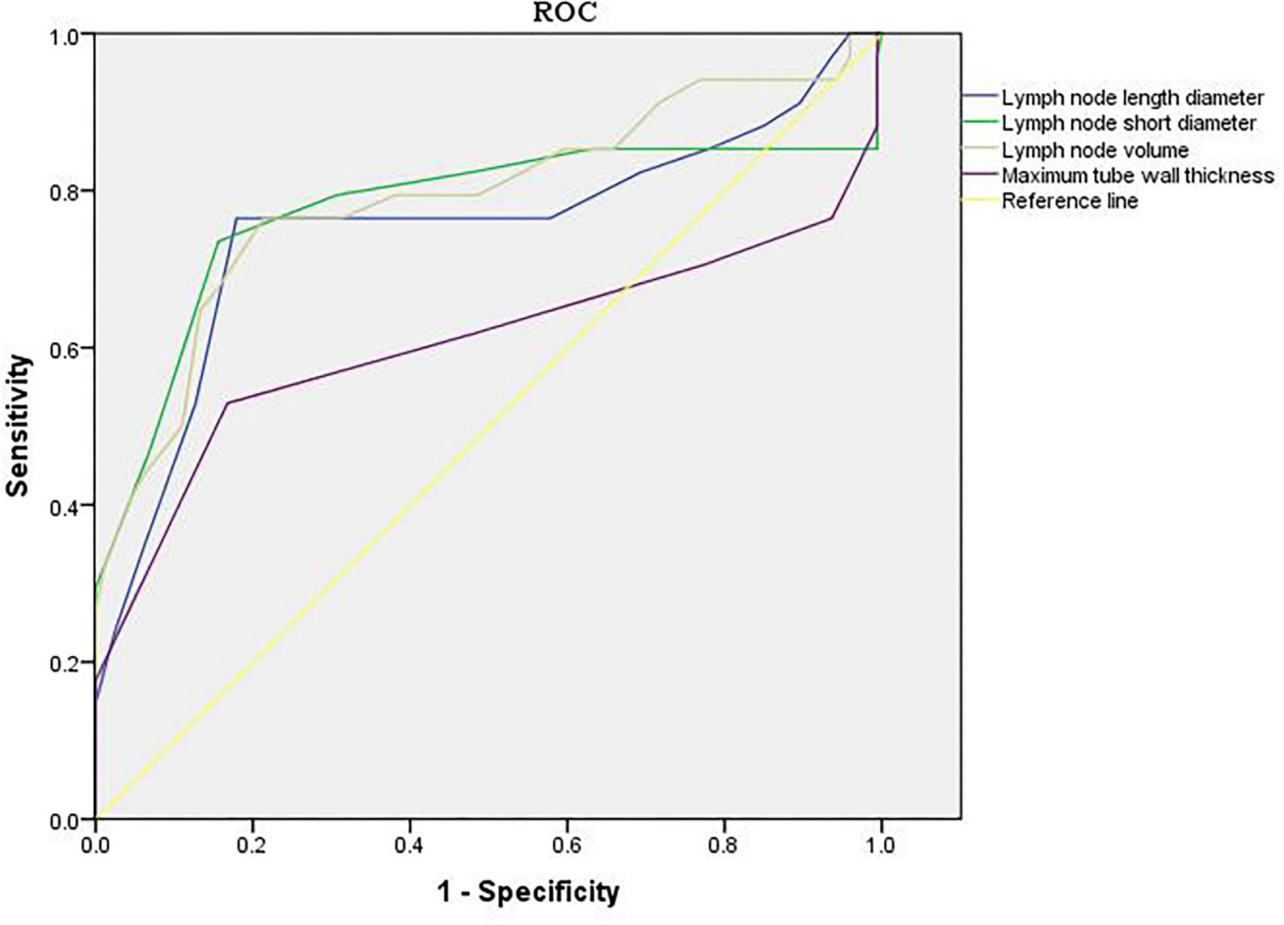
Value of imaging results in evaluating prognosis.

The AUC of length diameter, short diameter, and volume of the lymph node in evaluating the prognosis of patients with esophageal cancer was 0.751, 0.776, and 0.791, respectively (*p* < 0.05). The AUC of maximum tube wall thickness in evaluating the prognosis of esophageal cancer patients was 0.606 (*p* > 0.05) (as shown in Table 2 and Figure 5).

**Table 2:** Value of imaging results in evaluating prognosis.

**Index**	**AUC**	**Asymptotic 95% confidence interval**		**Standard error**	***P*-value**
		**Lower limit**	**Upper limit**		
Lymph node length diameter	0.751	0.639	0.862	0.057	0.000
Lymph node short diameter	0.776	0.660	0.893	0.059	0.000
Lymph node volume	0.791	0.692	0.891	0.051	0.000
Maximum tube wall thickness	0.606	0.468	0.743	0.070	0.052

## Discussion

In recent years, China's radiotherapy technology and equipment have made rapid development. From ordinary external irradiation to current three-dimensional conformal or intensity-modulated radiotherapy, it has become the main treatment of esophageal cancer. After radiotherapy, the local control rate and long-term survival rate of patients with esophageal cancer were improved compared with before radiotherapy (7, 8). However, it is still clinically necessary to accurately evaluate the short-term curative effect and prognosis of patients with esophageal cancer after radiotherapy, which has a positive role in improving the quality of life of patients.

Gastroenterography is a traditional examination method of digestive tract diseases, which is mainly used to observe digestive tract diseases, such as inflammation, ulcers, and tumors (9). The inspection principle of gastroenterography is: after the patient has taken a contrast agent, the reaction of the barium agent under the X-ray can be used to see the outline of the digestive tract and reveal the location of the disease (10). This examination is usually used for patients who cannot tolerate or cannot undergo gastrointestinal endoscopy. It has the advantages of relative safety, no side effects, and simple operation (11). Gastroenterography is widely used in clinical diagnosis and treatment. It is the most commonly used imaging examination method for esophageal cancer, and it is also the simplest examination method to evaluate the efficacy of radiotherapy. This examination method is highly sensitive to small lesions, mainly by observing mucosal folds to determine the location of esophageal lesions, to know the information on lesion size, growth form, and so on, and to reflect the changes in the esophageal cancer cavity (12, 13). However, gastroenterography can only evaluate the local remission of the primary tumor of esophageal cancer, and cannot effectively reflect the lymph node metastasis. Besides, there are some limitations in the evaluation of the wall thickness changes of esophageal cancer, and it cannot predict the prognosis more systematically (14).

At present, there are more and more studies on the combination of various imaging technologies for evaluating the curative effect and prognosis of tumors. As a technology characterized by displaying organ function and metabolic state, CT can not only observe the thickness of the esophageal wall and the interface at the wall before and after radiotherapy, but also determine the extent and degree of extra-esophageal cavity invasion, and the lymph node metastasis and distant metastasis can also be identified (15). CT is of great value in evaluating the extension of esophageal cancer. The esophagus lacks a serosa layer, and drainage lymph is unusually rich, so it is easy to directly invade adjacent organs and metastasis. There is a layer of fat around the esophagus, so CT can clearly show the relationship between the shape of the esophagus and the adjacent mediastinal organs below the esophagus (16, 17). CT scan directly measure the wall thickness, which is characterized by high resolution and simple operation. It can make up for the deficiency that gastroenterography can only observe local lesions in the esophageal cavity, and has higher accuracy in the evaluation of the short-term curative effect of radiotherapy, which is conducive to guiding clinical treatment (18). Gastroenterography combined with CT for comprehensive evaluation can complement each other's advantages and play a synergistic role in the evaluation of curative effect and prognosis of esophageal cancer.

Zhou's team found that the maximum diameter of metastatic lymph nodes may be an effective biomarker to predict the prognosis of patients who received radiotherapy after esophagectomy (19). Wu's team believed that the AUC for the minimum diameter of lymph nodes and the maximum diameter of lymph nodes for diagnosing lymph node metastasis were 0.679 and 0.666, respectively. CT can effectively evaluate lymph node metastasis in patients with thoracic esophageal cancer (20). In this study, after radiotherapy, the length diameter, short diameter, and volume of lymph nodes were lower than those before radiotherapy. The length diameter, short diameter, and volume of the lymph node in the good efficacy group and good prognosis group were lower, and the ORR in the good prognosis group was higher. The results showed that after radiotherapy, the smaller the length diameter, short diameter, and volume of lymph node, the more significant the short-term efficacy and the better the prognosis. The change of length diameter, short diameter, and volume of the lymph node is an important indicator to evaluate the risk of lymph node metastasis, and has a great influence on the therapeutic effect and prognosis.

Generally speaking, the tumor target area of radiotherapy for esophageal cancer is mainly based on the thickness of the esophageal wall (21). In this study, there was no significant difference in the maximum tube wall thickness before and after radiotherapy. The possible reasons are as follows: after radiotherapy, there are many changes in the inner mucosa of patients with esophageal cancer, such as edema, infiltration of inflammatory cells, congestion, erosion, and proliferation of connective tissue, granulation tissue, and collagen tissue in some places, which make it difficult for the thickness of the esophageal wall of patients to return to normal in a short time. Although some tumor cells were killed after radiotherapy, there was little change in the maximum tube wall thickness (22–24). Wu's team found that in patients with esophageal cancer undergoing radiotherapy and chemotherapy, the percentage of maximum wall thickness reduction was independently associated with pathological complete response (*p* = 0.027), and could predict the recurrence of the disease (25). Wongwaiyut's team found that the survival of patients with esophageal cancer with a maximum wall thickness of <10 mm and ≥10 mm was different, and the larger the maximum wall thickness, the worse the prognosis of patients (26). We found that the maximum tube wall thickness was lower in the good efficacy group and good prognosis group, but its value in evaluating the short-term efficacy and prognosis of patients with esophageal cancer was not high, and there was no significant difference. This indicated that the maximum tube wall thickness of patients with esophageal cancer is not related to short-term efficacy and prognosis, which may be due to the smaller maximum tube wall thickness of patients included in this study. In addition, the ROC curve of this study also found that the AUC of the length diameter, short diameter, and volume of the lymph node to evaluate the short-term efficacy of patients with esophageal cancer was 0.738, 0.705, and 0.748, respectively, and the AUC to evaluate the prognosis of patients with esophageal cancer was 0.751, 0.776, and 0.791, respectively. The results further showed that lymph nodes are closely related to the short-term efficacy and prognosis of patients with esophageal cancer. Gastroenterography combined with CT has a good application value in the evaluation of short-term efficacy and prognosis in patients with esophageal cancer radiotherapy. Conventional CT has higher requirements on the filling degree of the esophageal cavity. If the filling of the esophagus is not good, the display effect of CT will be affected to a certain extent. Medical personnel should pay attention to this during the detection process.

## Conclusion

To sum up, gastroenterography combined with CT has a good application value in the evaluation of short-term efficacy and prognosis in patients with esophageal cancer radiotherapy. There are still some deficiencies in this study. We only evaluated the 1-year survival rate of the patients, and the evaluation effect of gastroenterography combined with CT on the long-term prognosis of patients with esophageal cancer radiotherapy needs to be discussed in subsequent studies.

## Data Availability Statement

The original contributions presented in the study are included in the article/supplementary material, further inquiries can be directed to the corresponding author/s.

## Ethics Statement

This study was approved by the Ethics Committee of our hospital. All subjects gave informed consent and signed the informed consent form. The patients/participants provided their written informed consent to participate in this study.

## Author Contributions

LX is the executor and writer of the paper. LE is responsible for searching data and data analysis. DG is ensuring that the descriptions are accurate and agreed by all authors. ZW is responsible for research design. All the authors contributed to this article.

## Conflict of Interest

The authors declare that the research was conducted in the absence of any commercial or financial relationships that could be construed as a potential conflict of interest.

## Publisher's Note

All claims expressed in this article are solely those of the authors and do not necessarily represent those of their affiliated organizations, or those of the publisher, the editors and the reviewers. Any product that may be evaluated in this article, or claim that may be made by its manufacturer, is not guaranteed or endorsed by the publisher.
